# Utility and limitations of animal models for the functional validation of human sequence variants

**DOI:** 10.1002/mgg3.167

**Published:** 2015-08-03

**Authors:** Timothy C Cox

**Affiliations:** 1 Department of Pediatrics (Division of Craniofacial Medicine), University of WashingtonSeattle, Washington; 2 Center for Developmental Biology and Regenerative Medicine, Seattle Children’s Research InstituteSeattle, Washington; 3 Department of Anatomy & Developmental Biology, Monash UniversityClayton, Victoria, Australia

One of the biggest challenges facing us in this new age of genomic medicine is the functional validation of variants identified in exome/whole-genome sequencing approaches. The growing suite of bioinformatics tools provide powerful ways to filter and prioritize candidate genes as well as to offer predictions on the impact of coding variants on protein function. Yet, despite their usefulness in prioritizing candidates, these computational methodologies remain insufficiently robust to “prove” causation of, or contribution to, the phenotype of interest. When we serve as reviewers of manuscripts and grants we are often quick to remind authors of this fact and yet acknowledge that it is not always the easiest of tasks.

The need for caution over the reliance on bioinformatics is, however, justified. This of course is not to say these pipelines are not incredibly valuable in themselves. Indeed, they more often than not stimulate formulation of biologically testable hypotheses from which the evidence in support of causation may come. But the limitation is that almost all bioinformatics tools are built around existing knowledge that is acquired from public and/or commercial data sources and so inherit the inaccuracies and limitations of any past experimentation that generated that original data and, to an extent, are implicitly affected by errors in curation that are unavoidably present in such sources. Existing tools also lack the power to interpret biological context, including the complexities of developmental processes and tissue physiology that is currently the aim of the systems biologists. With the massive accumulation of “omics”-scale data, some of this will undoubtedly change. However, nothing is currently as convincing or as powerful as in vivo data from a good animal model. Even then, one must know the strengths and limitations of the model system being utilized and, equally importantly, have the tools by which to characterize the phenotypic outcome both appropriately and with precision. There is increasing recognition of the need for detailed and precise clinical phenotyping but sadly this has been lagging behind when phenotyping animal models. The latter problem is likely compounded by the pressures to publish, the cut backs and competitiveness in the current funding climate, and perhaps an under-appreciation of the insight provided by a well-characterized model and the translational impact that may be gleaned from it.

In this commentary, I wish to highlight the often difficult decisions we must face when confronted by a list of variants from genome-scale sequencing data and some key points to consider when deciding how to best test and validate any given variant as pathogenic.

## Familiar Scenarios

Consider the following scenarios that may be very familiar to many of you that have been lured to the promise of genomic nirvana. Note that the scenarios relate to sequencing of a relatively standard familial condition rather than of a cohort of singletons or even trios where the challenge can be even greater:

So, you have just received exome sequencing data on your family: data on three members, perhaps four depending on budget. Whether you performed the sequencing and/or bioinformatics yourself, or handed it to academic colleagues or one of the many industry providers, you now have an impressive spreadsheet detailing perhaps one or two dozen variants of interest that have survived the standard bioinformatics pipeline and meet the criteria of the presumed inheritance pattern in your family. With this “data” now residing on your hard-drive, excitement and optimism fills your head as you already envision the next big manuscript. But from hereafter, the story can go a number of ways:

### Scenario 1 [euphoria]

One gene in the filtered list stands out. It is a well-known gene from a well-characterized pathway. All affected members of the family harbor a nonsense change or at least a missense change in an evolutionarily conserved residue that can easily be predicted to disrupt its function. There are established in vitro assays or an available animal model. With some confidence, you can reasonably “ignore” the other genes harboring variants although you will, of course, report them in the manuscript’s supplementary data. The team outwardly rejoices.

### Scenario 2 [cautious optimism]

One or more genes harbor variants predicted to be damaging. Each of these genes has been implicated in pathways or cellular processes that could explain the clinical condition of interest although expression data indicates more widespread and variable levels of expression than desired for such a specific phenotype. There are some generic assays available, or you use what is clinically known about the condition to focus your investigations. You hope one, and only one, will reveal a deleterious impact on function within a relevant assay. You consider options for generating an animal model.

### Scenario 3 [depression]

Most of the genes in your “variant” list you have either never heard of or wished you had never heard of. From the few vague gene ontology (GO) terms associated with these genes, not even you can bring yourself to find a way to hypothetically link them to the family’s condition. As much as you wanted to find something unique and novel, a small (or perhaps large) part of you wanted a familiar gene so the functional validation experiments – that you know the reviewers will expect to see – will be logical. You begin to lament (a) the effort that went in to collect all those samples, (b) why you spent all that money from your hard earned grant for this outcome, (c) when (or whether) to tell your coinvestigators – perhaps miraculously you have missed something and all will resolve itself and save yourself the “shame”, and (d) how much this will impact on your impending competitive renewal application!

Unfortunately, scenarios 2 and 3, as well as other variations on the theme, are commonly encountered and likely to increase in occurrence as we try to resolve the genetic contribution to more and more conditions. So how did we get to this point and where do we go from here?

## The How and Where

Advances in genome sequencing technologies and the associated bioinformatics tools have clearly revolutionized genetics and brought genome-level sequencing data analysis within the realm of the ordinary researcher. The ease of access to, and rapidly reducing costs of, whole-exome sequencing (WES) has exponentially increased the available data on coding region and splice site variants yet there has not been a parallel increase in the ability to definitively discern pathogenicity. And this problem is significantly magnified with whole-genome sequencing (WGS) data where predicting, and in fact even detecting, potentially causal variants remains a computational challenge, let alone the task of providing biological evidence to support their involvement in the disease of interest.

Essential to maximizing the chances of a successful outcome in genome-level sequencing are robust phenotyping and strict clinical inclusion criteria (Allanson et al, 2009; Carey et al. 2012; Cox et al. 2013), although neither guarantees success. Inherently, we all accept phenotypic variability as a part of every human disorder and yet in many instances it seems the basis for this variability is ignored in favor of ensuring a larger cohort size perhaps under the falsehood that there will be increased resolving power in the genetic studies and the hope that reappearance of the same gene in more than one case will illuminate it as a likely causative gene. But crude phenotyping and larger cohorts can, in fact, have the opposite effect – reducing power and complicating identification of strong candidates.

The purposes of increased precision in phenotyping and strict classification criteria are to minimize genetic heterogeneity that can mask or at least reduce the certainty around identifying causal variants. The importance of careful phenotyping is nicely exemplified in the first report of the successful application of exome sequencing: the discovery of mutations in *MLL2* as a cause of Kabuki syndrome. In this case, the initial attempt at identifying the variant in exome sequencing data was unsuccessful. But careful reassessment of patient images and their clinical descriptions suggested adjustment to the bioinformatics pipeline, which proved crucial for finally finding the causal variants (Ng et al. 2010). Not only did this careful phenotyping lead to identification of the *MLL2* gene but it also supported the notion of genetic heterogeneity in the condition. Similar subphenotyping approaches have also been strongly encouraged in many other studies and conditions especially those where considerable genetic heterogeneity is assumed or already known, for example, cleft lip/palate (Jugessur et al. 2011).

One of the key aspects of accurate subphenotyping is the incorporation of quantitative (i.e., nonsubjective) measures, which typically requires well-defined normative values for the features being assessed. This move to quantitative assessment has been particularly evident in the fields of clinical genetics and dysmorphology. In fact, there is even an “app” to aid phenotyping for the dysmorphologist, called Face2Gene (http://www.fdna.com/face2gene/), which employs a facial recognition-like algorithm to identify facial gestalts characteristic of specific syndromes. However, like other bioinformatics tools, this program relies on input of facial images to build up a consensus facial “form” for each specific condition. Tools such as this, while exciting in their potential, rely on a sufficient repository of either genetically defined cases for each condition or large numbers of cases unambiguously diagnosed by an experienced dysmorphologist. In essence, similar digital tools could be developed in time to aid precision diagnosis of many clinical conditions as well as to standardize the phenotypic assessment of animal models.

With the advent of the new sequencing technologies, our experience shows that genetic heterogeneity is almost the norm, but arguably considerably greater than we could have predicted. Prominent examples include holoprosencephaly (14 genes plus additional chromosomal anomalies; Solomon et al., 2013), Bardet–Beidl syndrome (18 genes; M’hamdi et al. 2014), nephrolithiasis (30 genes; Halbritter et al. 2015), and Joubert syndrome (26 genes; Mancini et al. 2014). For these disorders, there are both dominant and autosomal forms, and yet still more genes remain to be found for each. While not all disorders will have this much heterogeneity, these conditions do highlight the many potential genetic inputs that not only determine but also influence the phenotypic presentation in any patient. This of course in turn raises considerable issues for genetic counseling. Again the example of cleft lip/palate is a useful one. Despite its high prevalence, the penetrance of cleft lip/palate as an isolated anomaly is low. Even in syndromes in which cleft lip/palate is a recognized feature and the genetic basis is known, the penetrance of overt clefting can vary from a few percent to as much as 60% or more with considerable variability in presentation even in members of the same family. Importantly, through extensive efforts by clinicians and researchers, many subclinical features are now known, including notches in the *orbicularis oris* muscle, distinctive lip whorl patterns and various dental anomalies (Marazita 2007; Neiswanger et al. 2009; Aspinall et al. 2014). Even the facial shape of “unaffected” parents of an affected child has been found to be distinctive (Weinberg et al. 2009). This increased phenotypic resolution over and above the segregation of different types of clefts (cleft lip only, cleft lip/palate, cleft palate only) has significantly aided the identification of associated gene variants in different populations. This more complete understanding of the phenotypic spectrum of specific conditions will ultimately be important as we move to more personalized medicine. But similarly, it should not be ignored in your animal model of choice. Animal models are far more amenable to extensive phenotypic investigations than patients and thus provide an opportunity not just to reproduce the main features of a disorder to provide strong evidence of causality of a gene but also to uncover more about the disorder of interest, especially for clinical conditions with marked genetic and phenotypic variability or rare conditions for which there are limited numbers of patients.

## Validating Variants not Candidate Genes

So whether you are fortunate enough to have a single attractive candidate gene from your sequencing efforts or many “vaguely possible” candidates, it is important to remember that the ideal goal is to validate the impact of the specifically identified variants and not just to provide support for the role of a selected candidate gene in a cell or developmental process that might explain the phenotype. Unfortunately, the former can be the most challenging and budget limiting. So, for most of us, generating multiple lines of evidence in support of a given candidate is the only feasible option.

The requirement for an animal model to provide support for a causal role for any candidate gene will depend on the nature of the genetic disorder under investigation. A disorder defined on the basis of a biochemical or metabolic deficiency for example may simply require assays in a cell culture system if the sole interest was to prove the variant(s) impacted the designated pathway activity. However, if one was interested in determining the impact of such a deficiency on development of the organism, for example if it was associated with characteristic dysmorphology or functional defects in patients, or a potential modifier or therapeutic intervention was sought, then an appropriate model system would be needed. In contrast, variants suspected of being causal for developmental, behavioral or physiological disorders cannot reasonably be validated outside of an *in vivo* context.

## Designing or Choosing the Best Animal Model

Whatever your animal model of choice, it is imperative that careful consideration is given to the most appropriate approach to modeling not just the most convenient. Below I discuss some of the more pertinent issues to consider using the mouse as the example model system. But many of these considerations are applicable to zebrafish and indeed now other model systems, especially given the rapid development of the relatively simple CRISPR/Cas technology (see later comments) to produce all types of genetic modifications including the insertion of loxP sites for conditional targeting and specific tags for tracking endogenous gene expression.

One of the primary considerations when generating or selecting a model is the nature of the gene variant under investigation. For example, will a straight gene knockout model truly mimic your human condition if you have a candidate with a missense mutation that is predicted to be damaging? Some experimental or computational investigation as to whether your variant is likely to be a complete loss of function versus a partial loss of activity could help significantly in deciding on the more appropriate approach to take. If your variant is expected to result in only a partial loss of protein function and your disease is recessive in nature, then a homozygous null animal model may exhibit a far more severe phenotype than you expect. In this case, you might consider assessing heterozygotes. Similarly, utilizing Cre-loxP technology to generate a conditional allele (i.e., one that can be deleted in a selected tissue or cell type and/or at a specific developmental time) can be a very powerful means to overcome any deleterious impact of eliminating your gene at all developmental stages and in all tissues and avoid complications because of expected earlier developmental roles for your gene. Importantly, a conditional-ready (or “floxed”) allele will still allow you to do a ubiquitous knockout via breeding with a Cre transgenic that expresses the Cre recombinase from a strong ubiquitous or “housekeeping” promoter, such as the *EIIa* promoter. However, one must also be careful in choosing Cre driver lines as many have been reported as tissue restricted but in reality also have other sites of activity that were not the focus of the original descriptive report and as such this can also sometimes impact the interpretation of your phenotype. Many Cre driver lines have been created via introduction of the Cre recombinase open reading frame at the initiation codon of an endogenous gene. The intent of this approach is to ensure the Cre is expressed in the same spatiotemporal context as the endogenous gene. However, this approach also results in loss of expression of the endogenous gene. Although these lines are often viable in the heterozygous state and appear “largely unaffected”, they are usually not viable as homozygotes or alternatively exhibit grossly visible phenotypes in the homozygous state. Investigations by a number of groups, including my own, have shown relevant phenotypes in heterozygotes for some Cre drivers created this way. But regardless, this loss of one allele of a gene that is expressed in the tissue or cell types that are of interest to your investigations can hypersensitize animals to disruptions in your candidate gene and thus exacerbate the phenotype and significantly impact interpretations. A prominent example of such a driver is the *Foxg1-Cre* line, which is frequently used to study the role of genes in the developing forebrain. *Foxg1-Cre* heterozygotes were initially reported as having no phenotype (Hébert and McConnell 2000), but it has since been shown that they in fact exhibit numerous developmental brain malformations on multiple different inbred backgrounds, with a particularly marked deficiency in the anterior telecephalon (Eagleson et al. 2007). Such issues should not exclude the use of these drivers, but the potential for such lines to confound interpretations needs to be appreciated by more researchers and the appropriate controls included in studies that utilize them.

An important consideration in your modeling approach should also be the mode of inheritance of the condition of interest. If your condition is dominant or semidominant (i.e., presents in heterozygotes), then assess heterozygous individuals in your animal model. Part of the issue with many animal model studies, particularly those performed by laboratories less experienced in their use or without the appropriate tools to phenotype the animals, is that descriptions of phenotypes in mutant mice tend to be quite cursory and often are only reported in homozygous null animals. While null animals can provide more easily visible phenotypes and “eye-catching” evidence of the role of a gene in the development of a given tissue, they do not mimic the typical human condition. For example, a homozygous null mutant can reveal a host of anomalies that are not seen (or at least are too mild to the naked eye) in the heterozygous state. Yet this does not necessarily provide evidence that this particular gene is responsible for your patient’s clinical presentation. A classic example of this is seen in mice carrying mutations in different genes that are located within the Williams–Beuren syndrome critical region. Original reports described mice homozygous null for the *Gtf2ird1* gene as having severe craniofacial defects and therefore the authors concluded that this gene is responsible for the typical facial gestalt of patients (Tassabehji et al. 2005). But patients with Williams–Beuren syndrome have heterozygous deletions of 7q11. Subsequent analysis of a hypomorphic allele of a second gene, *Baz1b*, also revealed significant craniofacial malformation in homozygotes while heterozygotes showed more subtle midfacial and posterior cranial changes akin to those seen in patients (Ashe et al. 2008). Which gene is truly responsible for the facial gestalt of Williams–Beuren syndrome is still not known as heterozygotes for the *Gtf2ird1* null allele were mentioned as being “unaffected” (sometimes a pseudonym for “no grossly visible abnormalities”). So either, both, or even yet another gene within the critical interval, could be responsible or contribute to the typical facial gestalt of patients. Nevertheless, the example serves to highlight the caution that must be taken when interpreting animal models.

In my own experience, I have lost count of the number of mouse models that were originally reported as not having a phenotype in heterozygotes but in which my lab or others has subsequently found significant (i.e., clinically relevant) phenotypes. And this list does not include the Cre driver issue mentioned above. Granted some of these phenotypes are less visually striking, but it is important to remember that many clinical conditions also require expertise for recognition of specific facial gestalts and/or other abnormalities. So why should an animal model be any different? Because investigators do not always have sufficient expertise in animal phenotyping or at least ready access to the appropriate tools and facilities, heterozygotes for many genes are frequently not studied and therefore many exciting animal models are potentially being overlooked. Contributing to this issue is the lack of specific “normative” measures or guidelines by which to assess one’s model organism of choice, just as was the case in humans until recently.

The general preference for generating severe or striking phenotypes may be an unfortunate by-product of the developmental biologists’ impact on the field of clinical genetics. From the perspective of a developmental biologist, striking phenotypes are desired because their interests typically lie in understanding the major genetic pathways regulating tissue morphogenesis. Many of these early morphogenetic events represent evolutionarily conserved processes that in turn are orchestrated by highly conserved gene pathways. These are not necessarily going to be the types of genes that will be responsible for the majority of human disorders, especially those with mild or modest clinical presentations. That said, there are certainly examples where mutations in evolutionarily important developmental pathways are prevalent, such as in components of the *SHH* pathway which underlie holoprosencephaly. These are typical disorders with more pronounced malformations. Nevertheless, the legacy inadvertently left is the all too common, but false, impression that if you cannot see, with the naked eye, a gross abnormality in your animal model then the phenotype is not important and therefore the gene is dispensable. I firmly believe that this is simply untrue and will continue to be borne out with the development of more sophisticated methods to analyze phenotypes in our animal models. In addition, phenotypes in mice can be highly variable, even on inbred genetic backgrounds, yet this variability in mice is not systematically documented like it is for patients in the medical genetics literature. This is despite the widespread acknowledgment that phenotypic presentations can be significantly influenced by epigenetic factors, and the realization that inbred mice are the ideal model system in which to tease out the contribution of such factors. These epigenetic influences are likely to be highly relevant for the development of effective personalized medicines and treatment plans.

Even though I am quick to espouse the many advantages offered by well thought out animal models, they are still not human and therefore their differences, and consequent limitations, also need to be appreciated and understood for phenotypes to be appropriately interpreted. Much can be gleaned from nonmammalian vertebrates such as zebrafish and chick, as well as invertebrate model systems such as *Drosophila*, *C.elegans* and yeast or one of a host of other unique systems. But their use must be relevant to the question being addressed and the data interpreted in light of the sometimes marked differences from humans. If questions are about gene or variant function on a genetic pathway within a cell or understanding the impact on tissue morphogenesis, then interpretation should be limited to such and not extrapolated to being “proof” or “evidence” to support a gene variant being causative. Even studies in mammalian model organisms such as the mouse still require an understanding of the differences in physiology, epidemiology, and developmental timing of events as this can sometimes have a large impact on disease susceptibility. In the ideal situation, introduction into your model organism of choice of the equivalent variant to that found in a patient would result in a homologous array of phenotypes. An excellent example is that seen in mice carrying the equivalent Fgfr2 mutation (S252W) that is commonly found in patients with Apert syndrome (Wang et al, 2005; Yin et al, 2008; Purushothaman et al. 2011) but there are many others.

## Other Options for Validating Variants

Bacteria and yeast were the original model systems for understanding gene function and important biochemical pathways relevant to human disease because they were the most amenable to genetic modification at the time. While they still play a key role in protein structural studies and pioneering proteomic technologies, they were superseded by invertebrate model systems such as *C. elegans* and *Drosophila melanogaster* which provided opportunities for cellular resolution studies of gene function in the context of multicellular developmental processes as well as means for high throughput screening for genetic mutants with specific cellular and developmental phenotypes. These models have been instrumental in understanding gene function and basic principles of cell signaling and interactions in coordinating morphogenetic processes and they still serve as valuable tools by which to understand basic evolutionarily conserved genetic pathways. However, over the past two decades – and particularly the last decade – the small zebrafish model system has taken over much of the role once dominated by *Drosophila* because it too offers the possibility of high throughput genetic screens, ease of access to embryonic time periods, and convenient phenotyping but in the context of a vertebrate organism. The recent development of simple genetic manipulation technologies, including shRNA/siRNA/miRNAs and, in particular, more recently the TALEN and CRISPR/Cas systems, has further boosted the power of the zebrafish system. However, in choosing to use this model system, one must still consider carefully the nature of the disease to be modeled and the subsequent phenotypic interpretation of any genetic manipulation because of the very different physiology, body structure, and even genome architecture (a partially trisomic genome).

The excitement over the CRISPR/Cas system, because of its speed and simplicity (and consequently the associated reduced costs of creating genetic modifications to investigate gene function and functionally test variants of interest *in vivo*) is almost palpable. Not only does this technology enhance the power of existing well-established models systems but it will also offer a means to generate precise modifications in many more model organisms that previously were not readily possible. Indeed, many Institutional transgenic facilities and industry providers are already offering the technology to their customer base to expand the options for generating mouse models. But like any new technology, there are still numerous kinks to be ironed out and further work needs to be done to ensure specificity of any targeted modification.

## Underutilized Resources for Animal Models of Human Genetic Disorders

In recent years, there has been a major push to expand the utilization of mouse models for studies aimed at understanding the pathogenesis of human genetic diseases. Many large internationally funded mutant mouse repositories offer thousands of superficially characterized models. For example, the Jackson Laboratories (JAX) in Maine is one of the largest of these resources, receiving and maintaining mutant lines originating from both individual investigators as well as the numerous international (i.e., KOMP) and ENU mutagenesis projects. The JAX also has their own in-house spontaneous mutant surveillance program from which many more mutants are derived, characterized, and stored for distribution. As with any large facility, phenotyping must be of moderately high throughput. Consequently, descriptions of specific phenotypes are often incomplete or fairly superficial yet in many cases sufficient to stimulate interest for further investigation. From my own experience of looking at dozens of mutant lines, those that arise spontaneously in their extensive breeding programs typically present with more mild and often-times more restricted or variable phenotypes than null mutations in the same genes created by traditional gene targeting approaches. As I mentioned earlier, I would argue that in many cases these represent better models of common human malformations than the many expensive targeted lines that have been generated. Nevertheless, one of the biggest hurdles in characterizing these lines is the lack of appropriate descriptors and strain-specific normative measures in mice although efforts are underway to improve this situation – much like the push in humans. The zebrafish community has also established a number of excellent repositories of mutant animals that can also be tapped for suitable models of candidate disease genes.

## Concluding Remarks

The usefulness of any given animal model is usually determined by how well the animal’s phenotype and/or disease progression is deemed to mimic that of the human condition. With the advanced tools for temporally and spatially controlling genetic manipulations, abundance of resources, and the opportunities to dissect out the contribution of both genetic and epigenetic/maternal factors, the mouse still remains the model organism of choice for understanding the pathogenesis of most human diseases. That said, other vertebrate models, in particular the zebrafish, certainly offer useful and very tractable systems for understanding the principles underpinning tissue development that lie at the heart of much human disease.

**Figure 1:**
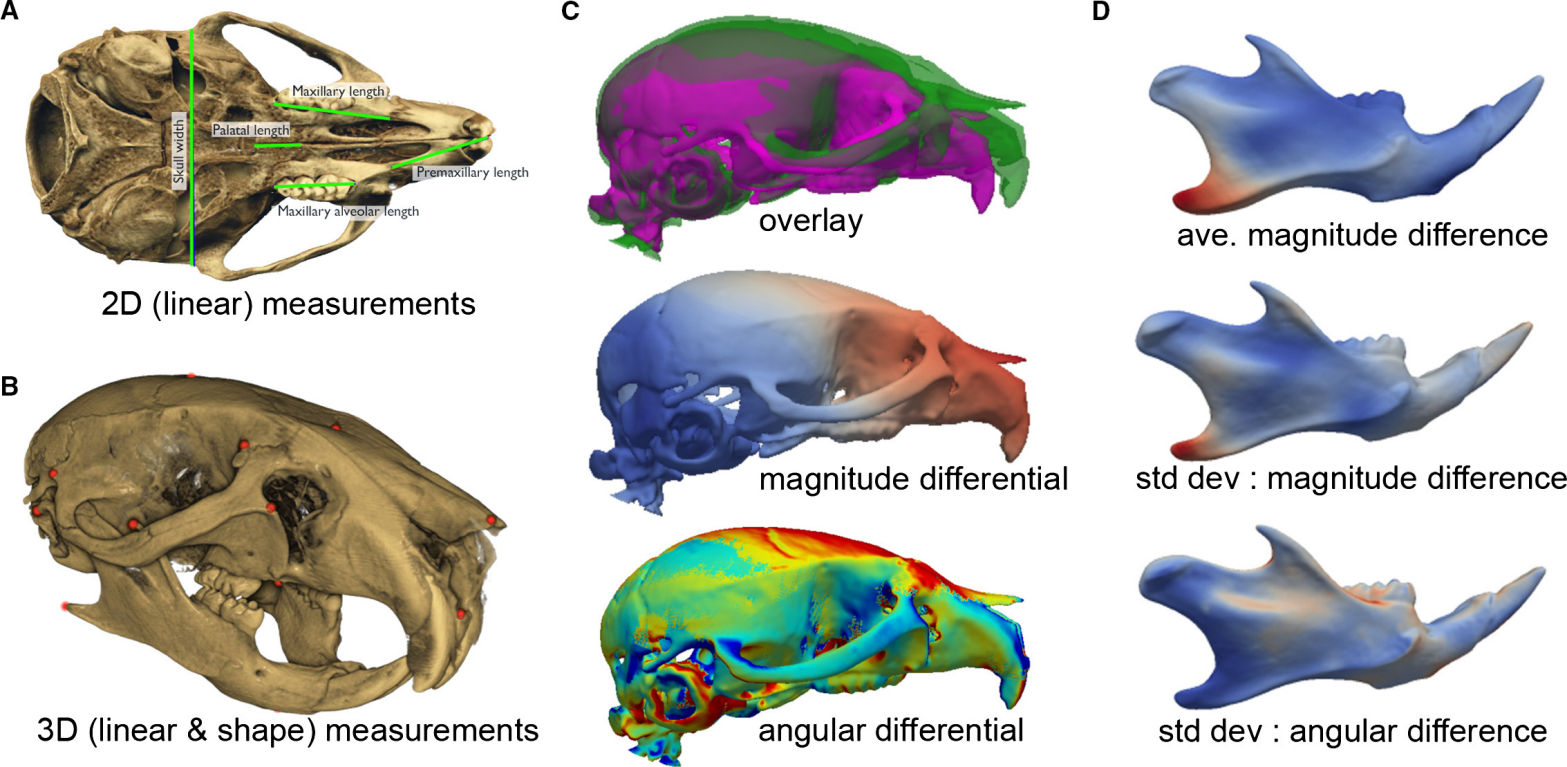
Examples of quantitative phenotyping in mouse models of specific human dysmorphologies. Examples shown are of different types of cranioskeletal analysis in mice but similar analyses can be performed on most tissues, organs, and model systems depending on the imaging modality chosen. (A) Linear measurements (green lines) from 2D images – the simplest form of quantitative assessment. (B) 3D linear measurements and morphometric shape analysis using 3D coordinates (red dots) collected from 3D imaging modalities such as microCT. Free software tools are available for this type of analysis and many core Institutional facilities offer access to advanced imaging modalities to capture 3D datasets for this type of investigation. (C) Types of analysis to compare individual specimens (e.g., mutant versus control). These assessments can include simple overlays of 3D renderings of each specimen (top – each mouse skull shown in a different color for ease of viewing), graphical representations of the magnitude (middle), or angular differences (bottom) between the skulls. The latter two are represented as “heat maps,” with the different colors denoting the scale of the differences from the control skull. (D) Types of analyses on groups of specimens, in this case mouse hemi-mandibles. The top image represents the average magnitude of the difference between mutants and control hemi-mandibles. Heat maps can also represent group statistics. In the middle and bottom images, respectively, the standard deviations of the magnitude differences and angular differences in the group of mutant animals are shown, which provide important information on phenotypic variation (Rolfe et al. 2013, 2014).

In molecular, biochemical, and even physiological studies, we have always expected data to be precisely quantified. As an example, decades ago RNA expression levels were quantified by determining the intensity of bands on a Northern blot. Then came qPCR technology and later the development of microarrays with a view to quantifying levels of mRNA on an increasing scale. And now we have RNAseq technology that is the ultimate in terms of quantifiable genome-scale data on RNA expression and splicing. In theory, this transitioning into “platforms” with more and more data comes with the expectation of greater precision in quantification. With the growing availability of high-resolution imaging capabilities (e.g., small animal magnetic resonance imaging [MRI] and micro-computed tomography [microCT]), technologies for automation, and tools for systems level analysis, we should be starting to expect precise and thorough quantitative phenotyping, including understanding the extent of phenotypic variability. Indeed many options are already available, both in commercial software packages and open-source or free software, to undertake such quantitative assessments of model organisms. Some of these tools do not require access to advanced imaging technologies (Fig.1A), while others maximize the enormous amount of data that is captured using these modalities (Fig.1B–D). In time, these tools and resources will become more readily available to – and manageable by – all researchers regardless of their level of expertise with the technology generating the raw data and the computational pipelines to output the data in an understandable format, much like the WES bioinformatics pipeline has in the last few years. So expect to see a new wave of technology coming your way soon – technology that will help focus our efforts on developing more accurate animal models of human genetic diseases and enable more precise interpretation of the effectiveness of potential treatments or interventional/preventative strategies for dealing with these conditions in patents.
